# Traditional Rehabilitation Experiences, Unmet Needs, and Perspectives on Virtual Reality–Based Rehabilitation Among Patients With Stroke in China: Qualitative Thematic Analysis and Semistructured Interview Study

**DOI:** 10.2196/84532

**Published:** 2026-02-02

**Authors:** Xite Zheng, Lu Xing, Haitao Lu, Shimeng Hao, Fen Liu

**Affiliations:** 1 Department of Epidemiology and Health Statistics School of Public Health, Beijing Key Laboratory of Environment and Aging Capital Medical University Beijing China; 2 School of Architecture and Urban Planning Beijing University of Civil Engineering and Architecture Beijing China; 3 Department of Neurorehabilitation Beijing Bo’ai Hospital, Rehabilitation Research Center, School of Rehabilitation Medicine Capital Medical University Beijing China

**Keywords:** stroke patients, rehabilitation, digital health technology, virtual reality, qualitative study, user-requirements, patient-centered design

## Abstract

**Background:**

Traditional stroke rehabilitation is facing challenges, and virtual reality (VR)–based rehabilitation is a promising solution. However, results from studies focusing on VR-based stroke rehabilitation remain inconsistent, largely due to the use of noncustomized interventions in previous trials.

**Objective:**

To enhance rehabilitation services and inform the development of patient-centered VR rehabilitation systems, this study aimed to (1) explore the experiences and unmet needs of survivors of stroke during current hospital rehabilitation, and (2) examine their perspectives on the use of VR technology in poststroke rehabilitation.

**Methods:**

We conducted a qualitative thematic analysis based on descriptive phenomenology between January and July 2025 at the China Rehabilitation Research Center. Adult patients with a clinical diagnosis of stroke within the past 18 months were eligible. A total of 21 survivors of stroke (mean age, 52.7, SD 17.3 y; men, n=17) were included. Data were collected through face-to-face semistructured interviews, complemented by a short questionnaire on sociodemographic, clinical, and technology-use characteristics. All interviews were audio-recorded, transcribed verbatim, and analyzed using a thematic approach, with thematic saturation used to determine the sample size.

**Results:**

After a stroke, patients experience significant physical and psychological changes. On the one hand, the sudden loss of abilities alters their perceived roles within the family and society; on the other hand, the sharp contrast between their desire for recovery and their current recovery limitations creates substantial psychological pressure. Accepting their condition and rebuilding confidence is a long-term process. Traditional rehabilitation is commonly described as burdensome, monotonous, and lacking continuity after discharge. Although patients desire a better rehabilitation approach and improved outcomes, attitudes toward VR-based rehabilitation vary. Some view VR as a convenient tool, while others express no interest or perceived need for technology-based rehabilitation. Patients indicated that serious games should be diversified to meet different individual and training needs, and should incorporate clearer feedback mechanisms, appropriate scoring systems, adjustable difficulty levels, and progressive game chapters. Functional expectations for VR systems included family involvement, access to personal rehabilitation data, telerehabilitation support, safety monitoring, and technical support.

**Conclusions:**

Stroke rehabilitation services in China require improvement in the appeal of rehabilitation content, patient self-management, and continuity of care. Although patients desire better rehabilitation approaches and outcomes, the effective integration of VR technology must account for factors, such as personal characteristics and preferences, as well as socioeconomic status. Unlike previous studies that primarily examined user experiences with digital technologies or compared rehabilitation outcomes, our research contributes to the literature by linking the challenges and patient needs in conventional rehabilitation with concrete directions for the future design of VR rehabilitation. These insights deepen current understanding of how VR technologies can be meaningfully integrated into stroke care and provide a roadmap for developing patient-centered and culturally responsive VR solutions.

## Introduction

Stroke is a leading cause of death and disability [1]. In 2021, there were an estimated 93.8 million survivors of stroke and 11.9 million new cases globally, including 26.3 million survivors and 4.1 million new cases in China [2]. Half of the survivors with stroke are left disabled, with one-third requiring assistance in daily activities [3]. In addition to physical impairments, cognitive and emotional disturbances, such as memory deficits, aphasia, and depression, are common and further reduce quality of life and hinder social reintegration [4]. Therefore, effective rehabilitation remains a critical component of stroke care in the long term.

While the rehabilitation research is moving toward the exploration of digital health technologies, current stroke rehabilitation in China primarily consists of physical therapy, occupational therapy, and speech therapy, with treatment plans tailored to individual conditions [5]. The demand for stroke rehabilitation services is increasing; however, substantial advances are yet to be made in stroke rehabilitation practice to meet this demand, including improving the effectiveness of current rehabilitation, enhancing adherence, and addressing limited rehabilitation resources, especially in home-based settings [6]. A deeper understanding of the real-world experiences and unmet needs of survivors of stroke throughout the rehabilitation process is critical for informing future service improvement and intervention development.

Virtual reality (VR)–based rehabilitation uses motion tracking, stereoscopic display, and real-time feedback to create immersive, interactive training environments, offering promising solutions for stroke rehabilitation [7]. Previous studies have shown that gamified training improves patient engagement and adherence [8]. Furthermore, the portability of the equipment and internet connectivity can improve access to treatment, promoting telerehabilitation, fostering equity, and supporting patient self-management [9,10]. VR-based rehabilitation has significant implications for improving current stroke care. Nevertheless, findings on its effectiveness remain mixed [11,12]. One major challenge is that many existing VR interventions rely on noncustomized content, such as commercial games, which fail to address the diverse functional needs, abilities, and preferences of survivors of stroke, ultimately limiting therapeutic effect [13]. Moreover, age, socioeconomic status, and educational differences influence individuals’ access to and familiarity with VR technology, further shaping intervention acceptability and outcomes [14]. The success of VR-based rehabilitation depends not only on technological efficacy but also on its alignment with the lived acceptability, preferences, and perceived needs of survivors of stroke. Therefore, listening to patients’ voices and developing patient-centered VR rehabilitation systems is crucial for addressing the current challenges [15].

Quantitative studies have provided important evidence on the effectiveness and feasibility of stroke rehabilitation interventions; however, they are limited in their ability to capture the rehabilitation experiences, unmet needs, and subjective perspectives of rehabilitation services among survivors of stroke. Qualitative approach and semistructured interviews were widely used to understand human phenomena, including patients’ thoughts and experiences [16,17]. Such information is essential for informing the development of patient-centered and culturally appropriate VR-based rehabilitation interventions.

Therefore, this study used qualitative methods to (1) explore the experiences and unmet needs of Chinese survivors of stroke during current hospital rehabilitation and (2) examine their perspectives on the use of VR technology in poststroke rehabilitation. The overarching goal is to enhance rehabilitation services and inform the development of patient-centered VR rehabilitation systems.

## Methods

### Research Design Overview

This study adopted a qualitative thematic analysis based on descriptive phenomenology to explore the rehabilitation experiences, unmet needs, and perspectives of survivors of stroke on VR-based rehabilitation [17,18]. Data were collected through semistructured interviews and analyzed using thematic analysis. This study was conducted at the China Rehabilitation Research Center (Beijing Boai Hospital), as part of a larger project entitled Development of a Motor and Cognitive Rehabilitation System for Stroke Patients Based on Multisensory Virtual Reality Technology.

### Researcher Description

This study was conducted through a collaboration among Capital Medical University, Beijing University of Civil Engineering and Architecture, and the China Rehabilitation Research Center. The research team consists of university professors, chief physicians, and doctoral and master’s students, all of whom have received professional training and possess extensive experience in stroke rehabilitation, qualitative research, and digital health. The primary interviewer and data analyst (XZ and LX) had received formal training in qualitative interviewing and thematic analysis, and possessed previous knowledge of stroke rehabilitation practices and the research landscape in China. XZ also had previous experience conducting and analyzing interviews with public health practitioners and primary and secondary school students. Reflexivity was maintained through regular team discussions, during which researchers reflected on their disciplinary backgrounds and potential assumptions to minimize interpretive bias. These experiences and foundation enabled the research team to successfully conduct this study and ensured the rigor of the methodology.

### Participants

The final sample included 21 participants (4 women and 17 men) with a mean age of 52.7 (SD 17.3) years. For most participants (19/21, 90%), the time since stroke onset was less than 6 months. The majority (14/21, 67%) reported daily internet use, and nearly all (19/21, 90%) owned a smartphone or tablet. Detailed participant characteristics are presented in Table 1.

**Table 1:** Demographic characteristics of patients with stroke at the Department of Neurorehabilitation, China Rehabilitation Research Center (January to July 2025, N=21, qualitative thematic analysis based on descriptive phenomenology).

Patient	Sex	Age (y)	Education level	Marital status	Time since stroke (months)	Stroke type	Perceived health	Smartphone or tablet	Use of the internet
P01	Female	62	Senior high or vocational school	Divorced	0-3	Ischemic	Good	Smartphone	Every day
P02	Male	90	Senior high or vocational school	Widowed	12-18	Ischemic	Fair	No	No
P03	Male	76	Junior high school	Married	0-3	Ischemic	Good	Smartphone	Every day
P04	Female	60	Junior high school	Married	0-3	Ischemic	Poor	No	No
P05	Male	46	Senior high or vocational school	Married	3-6	Ischemic	Fair	Smartphone	Multiple times a week
P06	Male	38	College, University, or above	Divorced	3-6	Ischemic	Fair	Both	Every day
P07	Male	58	Junior high school	Married	3-6	Hemorrhagic	Fair	Smartphone	Multiple times a week
P08	Female	69	Primary school or below	Married	0-3	Ischemic	Fair	Smartphone	Every day
P09	Male	55	College, University, or above	Married	0-3	Ischemic	Good	Smartphone	Every day
P10	Male	46	College, University, or above	Single	3-6	Hemorrhagic	Fair	Smartphone	Every day
P11	Male	30	Senior high or vocational school	Single	3-6	Hemorrhagic	Fair	Both	Multiple times a week
P12	Male	71	Senior high or vocational school	Married	3-6	Hemorrhagic	Good	Both	Multiple times a week
P13	Male	40	College, University, or above	Married	0-3	Ischemic	Fair	Both	Every day
P14	Female	27	College, University, or above	Married	6-12	Hemorrhagic	Fair	Both	Every day
P15	Male	62	Junior high school	Married	0-3	Hemorrhagic	Fair	Smartphone	Every day
P16	Male	38	College, University, or above	Married	3-6	Hemorrhagic	Fair	Smartphone	Multiple times a month
P17	Male	62	Junior high school	Married	3-6	Hemorrhagic	Fair	Both	Every day
P18	Male	32	College, University, or above	Single	0-3	Hemorrhagic	Good	Smartphone	Every day
P19	Male	32	College, University, or above	Married	0-3	Hemorrhagic	Fair	Both	Every day
P20	Male	70	College, University, or above	Married	0-3	Ischemic	Fair	Smartphone	Every day
P21	Male	43	College, University, or above	Married	3-6	Hemorrhagic	Fair	Smartphone	Every day

### Researcher–Participant Relationship

The researchers and participants had no previous relationship. At the scheduled interview time, a nurse introduced the researchers to the participants, and the participants felt at ease throughout the process.

### Recruitment Process

Physician-researchers screened medical records to identify eligible participants. Patients meeting the inclusion criteria were approached during hospitalization, and those who provided verbal consent were later contacted by trained interviewers to schedule a face-to-face interview. The sample size was determined by the principle of data saturation, defined as the point at which no new themes or insights emerged during analysis [19]. A total of 26 survivors of stroke were interviewed, of whom 5 were excluded due to poor communication ability, leaving 21 participants in the final analysis.

### Participant Selection

Participants were recruited through purposive sampling from the Department of Neurorehabilitation at the China Rehabilitation Research Center. Inclusion criteria were (1) age≥18 years, (2) first-ever stroke diagnosed within the past 18 months, and (3) ability to provide written informed consent. The 18-month time frame was chosen to capture both early and later recovery experiences and to examine the durability of these effects [20], while ensuring that participants were physically able to engage with the VR devices safely. To minimize unnecessary exclusion, patients with mild cognitive or communication impairments were permitted to receive support from informal caregivers when communication was slow. Exclusion criteria included inability to speak Chinese or insufficient communication capacity, as determined at the time of interview. Data collection took place between January and July 2025.

### Data Collection

Data were collected through face-to-face semistructured interviews following a predefined sequence. At the scheduled interview time, a nurse led 2 researchers (XZ and LX), both trained in qualitative research, to the patient’s bedside to confirm the appointment. After confirmation, the nurse left the room. The researchers then introduced themselves to the participant and explained the purpose and significance of the study. The participant then read and voluntarily signed the written informed consent. Participants were subsequently invited to complete 2 rounds of the Fruit Ninja (Halfbrick) game using a Pico 4 Enterprise headset (ByteDance; 1 round with visual access to the surrounding room environment and 1 without). Following the VR experience, participants completed a brief questionnaire collecting demographic and clinical information, including sex, age, marital status, education level, time since stroke, stroke type, self-perceived health, and use of mobile devices and the internet. Interviews were conducted after completion of the VR task and questionnaire, and took place either in the patient’s room or a doctor’s office to ensure a quiet, clean, and private environment. An interview guide (Multimedia Appendix 1), developed by the research team based on the study objectives and a review of relevant literature and refined through pilot interviews with 2 patients (data from the pilot interviews were not included in the final analysis), was used to facilitate the interviews. Interviews began with questions about stroke history and rehabilitation experiences, followed by exploration of perceived challenges, unmet needs, and suggestions for improving poststroke care. Participants were then asked to reflect on their VR experience, including attitudes, preferences, and perceptions of usability. Follow-up questions probed barriers to VR use and elicited recommendations for improvement. Open-ended prompts (“Is there anything else you would like to add regarding today’s topic?”) were used to encourage additional insights. Interviews lasted between 16 and 40 minutes (25 minutes on average), were audio-recorded, and transcribed verbatim.

### Data-Analytic Strategies

Data were analyzed using thematic analysis following Braun and Clarke’s 6-phase framework [21,22]. All interviews were transcribed within 24 hours by 2 researchers (XZ and LX). The first author completed a verbatim transcription, and the second author checked each transcript against the audio recordings to ensure accuracy and enhance familiarity with the dataset. Furthermore, the 2 researchers (XZ and LX) independently read and coded all of the transcripts using an inductive, data-driven approach, focusing on participants’ rehabilitation experiences, needs, and perspectives on VR technology. After independent coding, the 2 researchers compared their coding and discussed similarities and discrepancies. Discrepancies were resolved through in-depth discussion, during which the researcher explained their coding decisions with reference to the original transcripts. When consensus could not be reached initially, the relevant data segments were re-examined, and codes were refined or merged as appropriate until agreement was achieved. Codes were then organized into potential themes and subthemes, which were reviewed for consistency and relevance across transcripts. Recoding was performed when necessary. Themes were iteratively refined and finalized in consultation with the broader research team. All qualitative data were managed and analyzed using NVivo software (version 1.2; Lumivero). Selected quotations were translated into English using a forward-backward translation process.

### Methodological Integrity

Methodological integrity was ensured by aligning the study design, data collection, and analytic approach with the research aims. The study was conducted at the China Rehabilitation Research Center in Beijing, China, a leading institution with more than 30 years of experience in rehabilitation medicine and research, serving patients from across the country. A qualitative design informed by descriptive phenomenology was used to capture the rehabilitation experiences, unmet needs, and perspectives of survivors of stroke on VR-based rehabilitation. Semistructured interviews allowed participants to express their experiences, needs, and perspectives in their own words while ensuring coverage of key topics relevant to the research questions. Data collection continued until thematic saturation was reached, defined as the point at which no new themes emerged from successive interviews. Interviews were audio-recorded, transcribed verbatim, and analyzed using an inductive thematic analysis approach. Furthermore, 2 researchers (XZ and LX) independently coded the transcripts and engaged in iterative discussions to resolve discrepancies and refine the coding framework. Strategies to enhance methodological rigor, including reflexivity, trustworthiness, and ethical conduct, were integrated throughout the research process. These aspects are described in detail in the following sections: Researcher Description, Trustworthiness, and Ethical Considerations. This study adhered to the American Psychological Association’s reporting standards for qualitative research [23] and the COREQ (Consolidated Criteria for Reporting Qualitative Research) checklist (Multimedia Appendix 2) [24].

### Ethical Considerations

This study was reviewed and approved by the Medical Ethics Committee of Capital Medical University, Beijing, China (approval Z2025SY006). All procedures complied with the principles of the Declaration of Helsinki. Participants were informed of the study’s purpose, procedures, potential risks, and their right to withdraw at any time. Written informed consent was obtained before participation. To ensure privacy and confidentiality, data were deidentified through pseudonymization. Contact information was stored separately from research data. No images or personally identifiable information were included in the manuscript or supplementary materials. Participants received a small token of appreciation in the form of daily necessities valued at 20-30 Chinese yuan, equivalent to US $3-US $4.

### Trustworthiness

Trustworthiness was established following Guba and Lincoln’s criteria [25], encompassing credibility, transferability, dependability, and confirmability. Credibility was enhanced through timely verbatim transcription and cross-checking within 24 hours to ensure accurate and faithful representation of participants’ narratives. All interviews and analyses were conducted by 2 researchers (XZ and LX), facilitating deep engagement with the data. Transferability was supported by providing detailed descriptions of the study setting, recruitment context, and participant characteristics, enabling readers to assess the relevance of the findings to other contexts. Dependability and confirmability were strengthened through transparent documentation of data collection and analysis procedures. Data were independently coded by 2 researchers, with discrepancies resolved through discussion and consensus in consultation with the wider research team. Thematic analysis followed Braun and Clarke’s 6-phase framework, reducing potential researcher bias and enhancing analytic reliability.

## Results

### Overview

**Figure 1:**
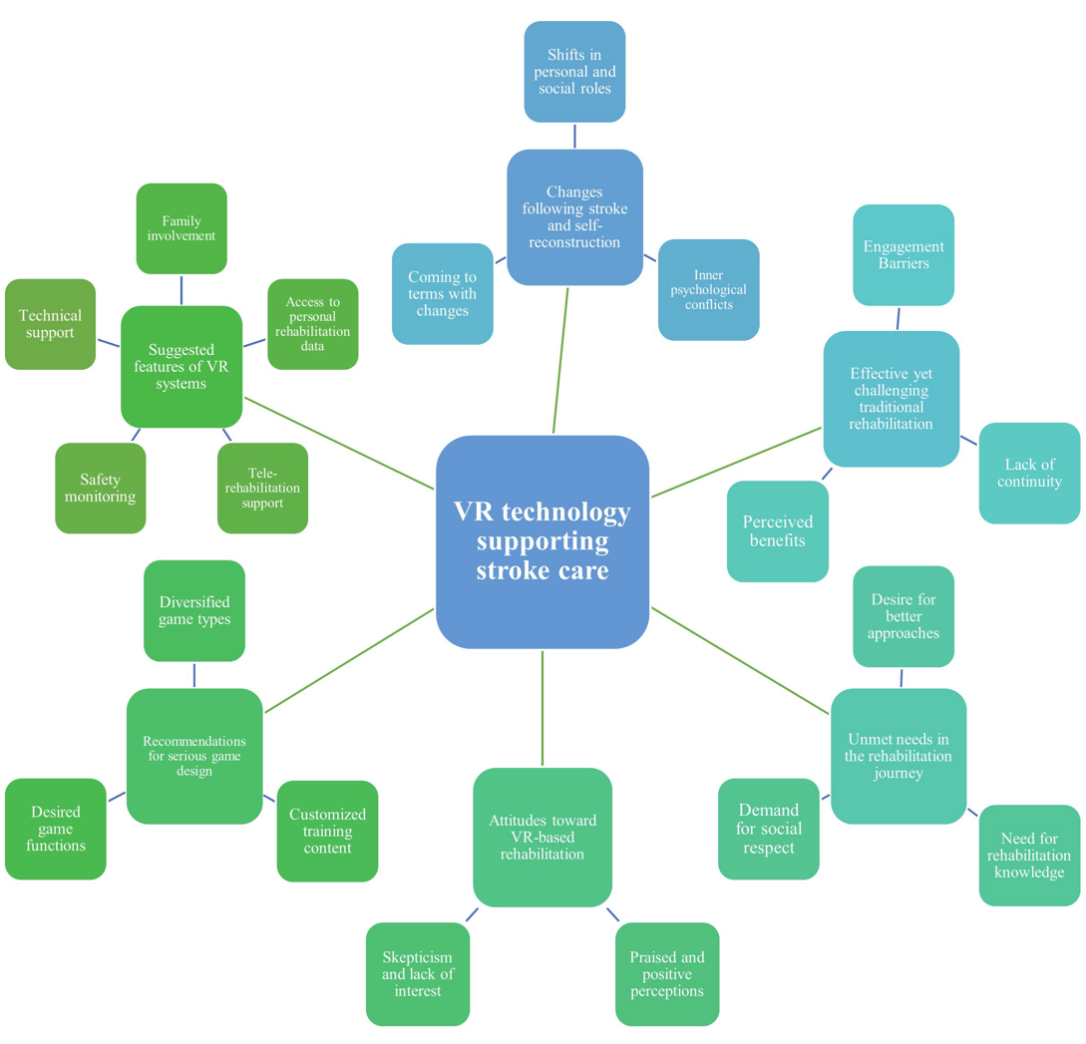
Themes and subthemes identified during data analysis of patients with stroke at the Department of Neurorehabilitation, China Rehabilitation Research Center (January and July 2025, N=21, qualitative thematic analysis based on descriptive phenomenology). VR: virtual reality.

Thematic analysis generated six overarching themes: (1) changes following stroke and self-reconstruction, (2) effective yet challenging traditional rehabilitation, (3) unmet needs in the rehabilitation journey, (4) attitudes toward VR-based rehabilitation, (5) recommendations for serious game design, and (6) suggested features of VR systems (Figure 1). A summary of themes and representative quotations is provided in Multimedia Appendix 3.

### Changes Following Stroke and Self-Reconstruction

#### Shifts in Personal and Social Roles

All participants experienced varying degrees of physical impairment following stroke. Some reported relatively mild deficits, such as limb weakness or fatigue, whereas others described severe hemiplegia accompanied by cognitive impairments, including confusion and aphasia. These functional losses led to substantial disruptions in their independence and resulted in major shifts in their roles within both personal and social contexts.

Overnight, my entire left side became paralyzed. I can’t do any work now. […] I’ve brought a lot of inconvenience to my family and close friends. That’s what pains me the most.

P09

Half of my body doesn’t move, so I rely on others for everything. At first, I couldn’t even speak or recognize people. I couldn’t do anything I was supposed to do. I became a burden to my family and brought misfortune to them.

P13

#### Inner Psychological Conflicts

The sudden loss of physical abilities and the associated role changes had profound psychological consequences. Participants described intense feelings of uselessness and a strong desire to recover bodily functions, which collectively created significant emotional pressure. Many reported experiencing anxiety, guilt, low self-esteem, irritability, and even despair.

After I got sick, I was constantly worried and sad. […] I felt irritable and depressed all the time.

P08

Right after it happened, I felt useless and even had thoughts of ending my life. But you cannot die even if you want to, because you cannot move. I couldn’t even pick up a knife to hurt myself. It was a feeling of utter despair.

P19

#### Coming to Terms With Changes

Despite these challenges, participants described conscious efforts to adopt a more positive mindset and gradually accept their condition. They highlighted the importance of patience, emotional stability, and realistic expectations to support long-term rehabilitation. Family encouragement and professional guidance played key roles in fostering acceptance and maintaining motivation.

Don’t rush it, take your time. Since you already have this illness, you need to stay calm. Whether it takes one or two years, even four, it is still the same condition. Recovery is slow, so you need patience. Being too anxious is not good for your recovery; once your mindset becomes tense, nothing goes well.

P18

Later on, with my family’s encouragement and the doctors’ support, I gained much more confidence in my recovery. You see, now I’m doing quite well.

P19

### Effective Yet Challenging Traditional Rehabilitation

#### Engagement Barriers

Many participants described current hospital rehabilitation as densely scheduled, repetitive, and lacking engagement. The monotony and high intensity of daily sessions often resulted in insufficient rest, leading to physical exhaustion and psychological strain. Over time, these factors diminished motivation, reduced adherence, and required substantial willpower to maintain participation.

The training is truly monotonous. I think it really depends on one’s willpower and endurance.

P05

My current rehabilitation includes OT, PT, robotic arm training, acupuncture, and massage. Except for massage, all the training is boring, just repeating the same movements every day.

P06

I have trained every weekday, only resting on weekends. My daughter had to push me in a wheelchair, and we are always rushing to get there on time; otherwise, I won’t be allowed to join the session. […] It’s exhausting. Sometimes my legs are swollen by the time I return to the ward.

P08

#### Lack of Continuity

Most participants reported performing little or no structured rehabilitation after hospital discharge. The absence of professional supervision, appropriate equipment, and adequate space at home posed major barriers to continuing rehabilitation outside the clinical environment.

At home, I can only go out for a walk and treat that as exercise.

P01

After I went home, I didn’t train at all. I think many people are like this. How can you train at home? There’s no equipment, no one to guide you, and no conditions for proper training.

P03

#### Perceived Benefits

Despite the challenges encountered, most participants expressed overall satisfaction with the effectiveness of rehabilitation. They also highlighted the emotional support provided by health care professionals, which contributed to their sense of comfort, motivation, and hope.

*The therapists here are very experienced. The environment in the rehabilitation department is alsoheartwarming. Someone will hold my hand or greet me kindly with comforting words, this really touches me.* [P02]

It’s been almost a month, and I’ll be discharged in a few days. My leg has improved a lot.

P03

During the rehabilitation process, the doctors constantly encouraged me, saying things like ‘You’re making progress’ or ‘You’re much better than a few days ago.’

P04

### Unmet Needs in the Rehabilitation Journey

#### Desire for Better Approaches

Several participants described transferring to the current hospital specifically to access more advanced technologies and effective rehabilitation interventions. Their accounts reflected a clear recognition of the limitations of previous treatments and a strong willingness to explore innovative strategies to optimize recovery.

This is already the third hospital I have been to. I started at the best hospital in my province, then went to the best local rehabilitation center, and now I am here at what they say is the top rehabilitation center in the country. I don’t really know what treatment methods they use here, so I feel the need to try and find out.

P09

#### Need for Rehabilitation Knowledge

Given the complexity and long duration of stroke recovery, many participants reported a lack of essential knowledge about their condition and appropriate rehabilitation strategies. They noted that their limited understanding of stroke mechanisms and treatment options hindered communication with health care providers and made it difficult to actively participate in decision-making.

We are not professionals, we don’t understand how this illness occurs or what the whole process involves. […] We truly lack knowledge. Now they are giving us these treatments, but we don’t know which ones are suitable for us. Even when doctors explain things, we still struggle to understand.

P10

#### Demand for Social Respect

Participants emphasized that social acceptance and respect are crucial for psychological resilience, well-being, and successful social integration. They noted that the support and treatment they receive vary greatly both within rehabilitation facilities and in society at large, and expressed a desire to receive the same level of respect outside of rehabilitation institutions.

When I came to this hospital, I noticed that some of the staff members are also people with disabilities like us. It shows we can still work and support ourselves even if we are disabled. This encourages me not to give up on life or my future. This is unlike the negative comments made by some people in society. The doctors and nurses are all very kind; they never show any prejudice because of our condition. Their respectful communication really helps with psychological healing.

P19

### Attitudes Toward VR-Based Rehabilitation

#### Praised and Positive Perceptions

Most participants generally expressed a positive attitude toward VR-based rehabilitation. They highlighted that the gamified and immersive nature of VR could make rehabilitation more engaging, increase motivation, and potentially enhance adherence compared with conventional, repetitive training. Participants also noted that VR could help address resource limitations after discharge and improve access by enabling rehabilitation outside the hospital environment.

Once this device is fully developed and widely promoted, it could be placed at the nurses’ station for patients to use in rotation. Patients could even purchase one to use at home.

P03

I think your VR device is very interesting, it’s completely virtual. My arm still feels a bit tired afterward, but the experience was enjoyable. […] Compared with the usual hospital treatments like cycling or traditional therapy, which are quite dull, this was much more engaging, though of course I can’t complain too much about hospital treatment either.

P20

#### Skepticism and Lack of Interest

In contrast, a subset of participants expressed hesitation or disinterest in using VR for rehabilitation. Many preferred conventional, face-to-face therapy due to its direct physical interaction and perceived reliability. Age-related limitations, physical discomfort, and economic concerns were also mentioned as barriers to adoption.

At my age, using computers is difficult. Sometimes I even feel dizzy.

P01

It depends on how much your device costs. I just tried it and my first impression was good, but if the price is too high, not everyone will be able to afford it, right?

P10

Honestly, I think what you are doing is great, but I just don’t think it’s necessary. No matter how good it is, I personally don’t need it.

P15

I still prefer traditional rehabilitation. It feels more intuitive—you can see it and touch it.

P17

### Recommendations for Serious Game Design

#### Diversified Game Types

Some participants suggested expanding the range of game genres, as people with different characteristics have different preferences. They emphasized that serious games should be tailored to users’ age, preferences, and rehabilitation stages. Incorporating culturally familiar or age-appropriate activities, such as calligraphy, chess, or dancing, was viewed as essential to ensure wider appeal and encourage maintained engagement.

I think you should design more types of games. I prefer games set in natural scenery and am not interested in this sword-based game.

P16

I think age differences matter. For people in their fifties or sixties, slicing fruit may not interest them. You could include chess or similar games that are more suitable for that age group.

P19

There are gender differences. Women might prefer dancing games.

P20

#### Customized Training Content

Although enjoyment was appreciated, participants emphasized that rehabilitation should remain the primary focus of serious games. They recommended developing targeted training modules that correspond to specific functional impairments and rehabilitation goals, such as upper limb strength, hand dexterity, or fine-motor tasks. They suggest creating a library of customizable exercises that clinicians could assign based on individual needs, ensuring both personalization and clinical relevance.

To me, muscle and strength training are most important because I want to walk independently. The game is great, and the virtual environment feels real, but I hope for more specialized training. For example, if I want to practice my hand, then exercises targeting finger movement or using chopsticks should be available. You could develop a game library with many options, and clinicians can select the ones that suit us.

P05

#### Desired Game Functions

Participants also proposed functional enhancements to improve the usability and therapeutic value of serious games. Adjustable difficulty levels and sequential game chapters were recommended to accommodate different impairment levels and to maintain long-term engagement. Scoring systems were considered valuable for monitoring progress and promoting self-motivation. Additionally, immersive environments and clear feedback, through audiovisual prompts, tactile feedback, or visual cues, were highlighted as necessary features, especially for individuals with sensory limitations.

Is the vibration on the controller too weak? I didn’t feel any vibration. You could add prompts or voice cues like ‘please touch’ or use arrows such as ‘move the controller here.’ That would be very helpful.

P04

I like a fully virtual environment because the scene feels realistic, and it gives you a sense of immersion. You’re not distracted by the outside environment, so you stay more focused during training.

P05

If you add a scoring system—like getting 1000 points today and 1500 tomorrow—I would feel like I’m making progress. By the third day, I’d aim for an even higher score. That kind of thing motivates players.

P10

Gradually increasing the difficulty, such as by speeding up or offering continuous stages, helps people adapt and stay interested in continuing.

P11

### Suggested Features of VR Systems

#### Family Involvement

Participants noted that VR rehabilitation devices resembled home gaming consoles, which made them especially appealing to family members, particularly children. Participants expressed a desire for family-connected gameplay, suggesting that allowing relatives to join the games could enhance enjoyment and strengthen emotional bonds during the recovery process.

This looks like a children’s game, [...] can my grandson play it?

P01

This is similar to the game consoles we have at home. Doesn’t Xbox have a game like this? […] I go home mainly to spend time with my daughter. She loves these kinds of games, so if we could play together, or if she could play with me in some way, that would be wonderful.

P21

#### Access to Personal Rehabilitation Data

Participants showed strong interest in accessing personal rehabilitation data directly through the VR system. They emphasized that personal medical and training records are crucial for understanding their treatment history, clarifying rehabilitation goals, and monitoring functional changes. Such transparency was viewed as essential for enhancing engagement, supporting self-management, and giving patients a sense of control over their rehabilitation journey.

Can you create a rehabilitation profile or progress tracking system? It looks like a game on the surface, but it’s actually rehabilitation training. You need to know what you’re training while playing and what you should do next.

P05

#### Telerehabilitation Support

Participants recommended integrating educational materials and professional guidance into the VR system to support rehabilitation outside the hospital. They recognized the potential of VR technology in providing remote access to medical services and ensuring continuity of rehabilitation.

If, during the rehabilitation process, doctors could see my progress and send me some rehabilitation-related suggestions, that would definitely be meaningful.

P02

The equipment in hospitals is very expensive and too large to use at home. If VR can be applied to home-based rehabilitation, I think it’s an excellent method. It’s lightweight, you can use it at home, and it still provides effective rehabilitation. That’s very good.

P19

#### Safety Monitoring

Safety emerged as a significant concern. Participants recommended incorporating physiological monitoring, such as blood pressure or fatigue indicators, and automated alerts to prevent overexertion or adverse events. They suggested implementing time limits, real-time feedback on training intensity, and emergency warnings. These proposals emphasize the need for built-in safeguards to protect vulnerable users, particularly older adults and those with cardiovascular conditions.

You have to limit the game time. What if someone plays all night? That would be dangerous.

P12

You could add some features focused on health monitoring. Since we mainly track blood pressure, you could include blood pressure measurements and provide specific values. That would make it medically useful and safer. Otherwise, someone might get too excited while playing and suddenly feel dizzy or faint. You could set an alert when blood pressure reaches a certain level, reminding the player to stop.

P19

#### Technical Support

Although participants found the current VR device easy to operate, many expressed concerns about potential technical issues or malfunctions, especially when used at home. They emphasized the need for accessible customer support and reliable maintenance to ensure confidence in long-term use. Suggested options included 24-hour service hotlines, troubleshooting through widely used communication platforms such as WeChat (Tencent Holdings Limited), and guaranteed repair.

If I buy it and take it home, I need help when the device has problems, something like a customer service number or a WeChat account.

P03

If I am going to use it long-term, it needs to have warranty services. If it breaks, someone must be responsible for repairing it.

P12

## Discussion

### Principal Findings

This qualitative study explored the experiences and unmet needs of survivors of stroke during current hospital rehabilitation, as well as their perspectives on integrating VR technology into stroke rehabilitation. Thematic analysis revealed six overarching themes: (1) changes following stroke and self-reconstruction, (2) effective yet challenging traditional rehabilitation, (3) unmet needs in the rehabilitation journey, (4) attitudes toward VR-based rehabilitation, (5) recommendations for serious game design, and (6) suggested features of VR systems. Unlike previous studies that primarily examined user experiences with digital technologies or compared rehabilitation outcomes, our research contributes to the literature by linking the challenges and patient needs in conventional rehabilitation with concrete directions for the future design of VR-based rehabilitation.

Survivors of stroke experience profound shifts in their roles within families and society, and the stark contrast between their desire for recovery and the harsh realities of impairment generates substantial psychological pressure. Support from family members and health care professionals plays a critical role in helping patients regain confidence and rebuild their lives. Traditional rehabilitation programs are often described as burdensome and monotonous, and the near absence of home rehabilitation presents another major challenge. Although patients desire more engaging rehabilitation approaches and better functional outcomes, future VR rehabilitation devices must account for differences in personal characteristics and preferences, as well as socioeconomic factors. Moreover, to maximize both effectiveness and acceptability, VR rehabilitation systems should incorporate serious games tailored to diverse personal and training needs, and integrate functions such as family interaction, progress tracking, telehealth support, safety monitoring, and technical assistance.

### Comparison With Previous Work

The level of exercise rehabilitation among survivors of stroke remains far below guideline recommendations, often characterized by the “three lows”: low initiative, poor adherence, and reduced willingness to participate [26]. From a Chinese cultural perspective, rehabilitation often relies on rest or passive treatments, such as the concept of “Deqi” in traditional Chinese medicine [27], which may weaken motivation for active, repetitive training. Furthermore, access to rehabilitation services remains uneven across regions, and high out-of-pocket costs further reduce opportunities for maintained participation [28]. In our study, traditional rehabilitation exercises were frequently described as burdensome, monotonous, and lacking continuity. Previous studies have shown that repetitive movements without feedback can diminish motivation among patients with stroke [29]. After hospital discharge, many participants experienced a sharp decline in available resources and limited professional guidance [30]. In contrast, VR-based rehabilitation is reported to be more engaging than traditional face-to-face therapy with a therapist [31]. The portability of VR devices and the gamified approach to intervention can enhance motivation and extend rehabilitation into the community and home environments, thereby improving continuity of care [32].

Consistent with previous studies, survivors of stroke in our sample expressed varying attitudes toward VR-based rehabilitation [33]. Particularly in the context of traditional Chinese culture, many individuals still view games as purely for entertainment, which may lead to skepticism about game-based rehabilitation. This underscores the importance of individual readiness, digital literacy, and personal preferences in shaping acceptance and effectiveness of VR-based interventions. Notably, even participants who were initially skeptical of VR acknowledged the value of advanced hospitals and innovative rehabilitation technologies and expressed a desire for improved rehabilitation programs. Previous research indicates that patients are more likely to adopt digital health tools when they are simple, intuitive, and user-friendly [34], whereas poorly designed equipment can lead to negative experiences and reduced adherence [35]. Importantly, firsthand experience with the benefits of digital health technologies plays a key role in promoting acceptance and maintained use [36]. Therefore, when implementing VR in clinical practice, it is essential not only to clearly communicate the practical benefits of VR-based rehabilitation but also to address patients’ concerns related to usability, safety, and reliability.

Serious games have been increasingly evaluated in clinical rehabilitation protocols, with studies reporting improved patient engagement and functional outcomes [37]. However, understanding how patients with diverse characteristics perceive different game elements is essential for maximizing acceptance, adherence, and therapeutic effectiveness. In our study, participants emphasized the importance of offering game types aligned with personal interests and selecting training content tailored to individual needs. In addition, due to decreased reaction times and slower cognitive processing, serious games should incorporate clearer feedback mechanisms, appropriate scoring systems to enhance motivation, and adjustable difficulty levels with progressively structured game chapters to accommodate varying motor abilities.

Survivors of stroke in our study also identified several desirable features for VR-based rehabilitation systems, including family involvement, access to personal rehabilitation data, telerehabilitation support, safety monitoring, and technical assistance. Family engagement has long been shown to enhance motivation, emotional support, and adherence in stroke rehabilitation [38]. Participants highlighted that family support from family members is critical for psychological healing, yet complex emotional challenges are often overlooked despite their substantial influence on rehabilitation outcomes [39]. A VR gaming platform that allows family members to participate together can, while providing entertainment, strengthen the bonds between family members and even promote intergenerational communication. Additionally, progress tracking can enhance self-efficacy, support goal-setting, and promote active self-management [40]. In our study, many participants expressed strong enthusiasm about VR’s potential to increase engagement, maintain motivation, and improve accessibility, particularly for home-based rehabilitation. In a home setting, patients can independently complete structured therapeutic exercises through VR tutorials delivered via head-mounted displays and controllers [41,42]. Remote therapist supervision can be supported through real-time data transmission, video consultations, or automated progress reports generated by the system [43]. This potential became particularly evident during the pandemic, when remote technologies played an essential role in maintaining continuity of care. Built-in safety features, such as heart rate and blood pressure monitoring, usage timers, and fall detection alarms, can further enhance user motivation and reduce risks during home use [44]. Finally, reliable technical support is crucial for enhancing patient trust and ensuring long-term adherence.

The successful integration of VR technology into stroke rehabilitation, as well as equitable access to such innovations, requires not only technological advancement but also governmental support and a cultural shift within clinical practice toward embracing digital rehabilitation tools. Achieving this goal calls for interdisciplinary collaboration among rehabilitation clinicians, neuroscientists, engineers, designers, and patients to develop evidence-based systems and clinical protocols that ensure both effectiveness and usability [45]. Special attention should be given to adapting VR interventions for diverse populations, particularly older adults with limited digital literacy and individuals living in resource-constrained environments. Critical challenges, including digital literacy, cost, security, and accessibility in rural areas [14,46], may be addressed through 2 complementary pathways: designing simple, intuitive systems supported by clinical validation, and reducing production costs through governmental funding while implementing pilot programs in community and rural settings. Additionally, VR systems must account for the evolving priorities of survivors of stroke throughout their recovery [47]. A phased, holistic intervention approach is needed to support the restoration of physical, psychological, and social functions. Using Maslow’s hierarchy of needs as a conceptual framework [48,49], VR system design should adapt its focus at different recovery stages. In the early phase, rehabilitation focuses on stabilization, preventing complications, and gently initiating motor function while offering psychological support. In the subacute phase, it shifts to intensive task-specific training and cognitive and motivational interventions. In the long-term community or home setting, rehabilitation emphasizes daily-life reintegration, supported by family involvement, peer support, and continued physical and psychosocial training.

### Strengths and Limitations

The strengths of this study include allowing participants to experience VR rehabilitation equipment before each interview, which enhanced their understanding and enabled them to provide more informed feedback. Additionally, we implemented rigorous qualitative methods appropriate for an exploratory study, allowing patients to express their rehabilitation experiences, unmet needs, and perspectives on VR freely and comprehensively. However, several limitations should be acknowledged. First, the sample was drawn from a single rehabilitation hospital and was predominantly male, which may limit generalizability. Second, all interviews were conducted in Chinese and subsequently translated into English, which may have introduced subtle linguistic or interpretive biases. Third, most participants were within 6 months of stroke onset, which may restrict insights into long-term or home-based rehabilitation experiences. Fourth, the relatively short interview duration and insufficient depth in probing questions may have constrained the richness of the data. Finally, the absence of longitudinal follow-up prevented examination of how patients’ needs, self-management behaviors, and acceptance of VR may change over time. Future research should incorporate multicenter longitudinal studies with more diverse samples to explore how patients’ needs and attitudes evolve across recovery stages.

### Conclusions

This study explored the rehabilitation experiences and unmet needs of survivors of stroke, as well as their perspectives of VR-based rehabilitation. Survivors of stroke undergo significant changes in their family and social roles due to the sudden loss of physical abilities. Their strong desire for recovery stands in stark contrast to the slow progress they experience in reality, which causes them immense psychological stress. Traditional rehabilitation is described as arduous and monotonous, with little to no support for home-based rehabilitation. Consequently, stroke rehabilitation services in China require improvements in the attractiveness of rehabilitation content, patient self-management, and continuity of care. Although patients desire better rehabilitation approaches and outcomes, the effective integration of VR technology into stroke care must take into account factors, such as personal characteristics and preferences, as well as socioeconomic status. Future VR rehabilitation systems should incorporate serious games that address diverse personal and training needs and integrate functions, such as family involvement, progress tracking, telemedicine support, safety protection, and technical assistance. These findings deepen understanding of how VR technologies can be meaningfully embedded into stroke rehabilitation and offer a roadmap for developing patient-centered and culturally responsive VR solutions.

## Appendix

Multimedia Appendix 1 Sample Interview Guide.

Multimedia Appendix 2 COREQ checklist.

Multimedia Appendix 3 Summary of Themes and Representative Quotations.

## Abbreviations

VR: virtual reality
COREQ: Consolidated Criteria for Reporting Qualitative Research

## Data Availability

The data supporting this study are available upon reasonable request and subject to approval by the corresponding authors. Requests should be directed to SH and FL.
