# Supplementary material for: Traditional Rehabilitation Experiences, Unmet Needs, and Perspectives on Virtual Reality–Based Rehabilitation Among Patients With Stroke in China: Qualitative Thematic Analysis and Semistructured Interview Study
Source: J Med Internet Res. 2026 Feb 2;28:e84532. doi: 10.2196/84532 (PMC12910270; doi:10.2196/84532)
Supplement: Multimedia Appendix 1 [file jmir_v28i1e84532_app1.docx]

**Multimedia Appendix 1: Sample Interview Guide**

**Introduction**

• Briefly introduce the background and purpose of the study, and explain the structure and estimated duration of the interview.

• Confirm that the participant has provided informed consent and granted permission for audio recording.

**Experiences and Needs in Stroke Rehabilitation**

• Can you describe how your life has changed since experiencing a stroke?

• What rehabilitation treatments are you currently receiving or have you received in the past?

• What challenges have you encountered during your recovery process?

• What kinds of support or resources do you think could help improve your rehabilitation?

• According to you, what is important and/or valuable for stroke patients regarding treatment and care?

**Perspectives on Virtual Reality (VR) in Rehabilitation**

• Based on your experience using the virtual reality equipment, how did you feel about it overall?

• Do you think virtual reality is a good option for stroke rehabilitation? Why or why not?

• What potential challenges or barriers do you foresee in using VR technologies?

• In your opinion, what features or characteristics should a VR rehabilitation system have to make it suitable and easy for you to use?

• Do you have any suggestions for improving VR-based rehabilitation tools or systems?

• Is there anything else you would like to add regarding today’s topic?

**Closing**

• Express sincere appreciation for their time and contribution.
