# Supplementary material for: Traditional Rehabilitation Experiences, Unmet Needs, and Perspectives on Virtual Reality–Based Rehabilitation Among Patients With Stroke in China: Qualitative Thematic Analysis and Semistructured Interview Study
Source: J Med Internet Res. 2026 Feb 2;28:e84532. doi: 10.2196/84532 (PMC12910270; doi:10.2196/84532)
Supplement: Multimedia Appendix 2 [file jmir_v28i1e84532_app2.docx]

**Multimedia Appendix 2: Completed Consolidated Criteria for Reporting Qualitative Research (COREQ) checklist.**

| **DOMAIN** | **ITEM** | **GUIDE QUESTIONS / DESCRIPTIONS** | **REPORTED IN SECTION** |
| --- | --- | --- | --- |
| **DOMAIN 1: RESEARCH TEAM AND REFLEXIVITY** | | | |
| ***Personal characteristics*** | | | |
| Interviewer/facilitator | 1 | Which author/s conducted the interview or focus group? | Researcher Description |
| Credentials | 2 | What were the researcher's credentials? (e.g., PhD, MD) | Title page |
| Occupation | 3 | What was their occupation at the time of the study? | Researcher Description |
| Gender | 4 | Was the researcher male or female? | Except for Xite, all are women |
| Experience and training | 5 | What experience or training did the researcher have? | Researcher Description |
| ***Relationship with participants*** | | | |
| Relationship established | 6 | Was a relationship established prior to study commencement? | Researcher–Participant Relationship |
| Participant knowledge of the interviewer | 7 | What did the participants know about the researcher? (e.g., personal goals, reasons for doing the research) | Data collection |
| Interviewer characteristics | 8 | What characteristics were reported about the interviewer/facilitator? (e.g., bias, assumptions, reasons, and interests in the research topic) | Researcher Description |
| **DOMAIN 2: STUDY DESIGN** | | | |
| ***Theoretical framework*** | | | |
| Methodological orientation and theory | 9 | What methodological orientation was stated to underpin the study? (e.g., grounded theory, discourse analysis, ethnography, phenomenology, content analysis) | Research Design Overview |
| ***Participant selection*** | | | |
| Sampling | 10 | How were participants selected? (e.g., purposive, convenience, consecutive, snowball) | Participant Selection |
| Method of approach | 11 | How were participants approached? (e.g., face-to-face, telephone, mail, email) | Recruitment Process |
| Sample size | 12 | How many participants were in the study? | Recruitment Process |
| Non-participation | 13 | How many people refused to participate or dropped out? Reasons? | Recruitment Process |
| ***Setting*** | | | |
| Setting of data collection | 14 | Where was the data collected? (e.g., home, clinic, workplace) | Data collection |
| Presence of non-participants | 15 | Was anyone else present besides the participants and researchers? | Data collection |
| Description of sample | 16 | What are important characteristics of the sample? (e.g., demographic data, date) | Participants; Table 1 |
| ***Data collection*** | | | |
| Interview guide | 17 | Were questions, prompts, guides provided by the authors? Was it pilot tested? | Data collection |
| Repeat interviews | 18 | Were repeat interviews carried out? If yes, how many? | No repeat interviews were carried out. |
| Audio/visual recording | 19 | Did the research use audio or visual recording to collect the data? | Data collection |
| Field notes | 20 | Were field notes made during and/or after the interview or focus group? | Data-Analytic Strategies |
| Duration | 21 | What was the duration of the interviews or focus group? | Data collection |
| Data saturation | 22 | Was data saturation discussed? | Recruitment Process |
| Transcripts returned | 23 | Were transcripts returned to participants for comment and/or correction? | No; however, timely verbatim transcription and verification within 24 hours to ensure accurate and faithful representation of participants’ expressions. |
| **DOMAIN 3: ANALYSIS AND FINDINGS** | | | |
| ***Data analysis*** | | | |
| Number of data coders | 24 | How many data coders coded the data? | Data-Analytic Strategies |
| Description of the coding tree | 25 | Did authors provide a description of the coding tree? | Figure 1; Appendix 3. |
| Derivation of themes | 26 | Were themes identified in advance or derived from the data? | Data-Analytic Strategies. Themes were identified from the data. |
| Software | 27 | What software, if applicable, was used to manage the data? | Data-Analytic Strategies |
| Participant checking | 28 | Did participants provide feedback on the findings? | No. |
| ***Reporting*** | | | |
| Quotations presented | 29 | Were participant quotations presented to illustrate the themes/findings? Was each quotation identified? (e.g., participant number) | Results; Appendix 3. Data were de-identified. |
| Data and findings consistent | 30 | Was there consistency between the data presented and the findings? | Results. Data presented and findings are consistent. The quotes explicate the themes as presented. |
| Clarity of major themes | 31 | Were major themes clearly presented in the findings? | Results. |
| Clarity of minor themes | 32 | Is there a description of diverse cases or discussion of minor themes? | Results. We described different cases and used representative quotes. |
