# Supplementary material for: Traditional Rehabilitation Experiences, Unmet Needs, and Perspectives on Virtual Reality–Based Rehabilitation Among Patients With Stroke in China: Qualitative Thematic Analysis and Semistructured Interview Study
Source: J Med Internet Res. 2026 Feb 2;28:e84532. doi: 10.2196/84532 (PMC12910270; doi:10.2196/84532)
Supplement: Multimedia Appendix 3 [file jmir_v28i1e84532_app3.docx]

**Multimedia Appendix 3: Summary of Themes and Representative Quotations.**

| **Theme and subtheme** | **Example quote** |
| --- | --- |
| **Changes following stroke and self-reconstruction** | |
| Shifts in personal and social roles | Overnight, my entire left side became paralyzed. I can’t do any work now. […] I’ve brought a lot of inconvenience to my family and close friends. That’s what pains me the most. (P09)  Half of my body doesn’t move, so I rely on others for everything. At first, I couldn’t even speak or recognize people. I couldn’t do anything I was supposed to do. I became a burden to my family and brought misfortune to them. (P13) |
| Inner psychological conflicts | After I got sick, I was constantly worried and sad. […] I felt irritable and depressed all the time. (P08)  Right after it happened, I felt useless and even had thoughts of ending my life. But you cannot die even if you want to, because you cannot move. I couldn’t even pick up a knife to hurt myself. It was a feeling of utter despair. (P19) |
| Coming to terms with changes | Don’t rush it, take your time. Since you already have this illness, you need to stay calm. Whether it takes one or two years, even four, it is still the same condition. Recovery is slow, so you need patience. Being too anxious is not good for your recovery; once your mindset becomes tense, nothing goes well. (P18)  Later on, with my family’s encouragement and the doctors’ support, I gained much more confidence in my recovery. You see, now I’m doing quite well. (P19) |
| **Effective yet challenging traditional rehabilitation** | |
| Engagement Barriers | The training is truly monotonous. I think it really depends on one’s willpower and endurance. (P05)  My current rehabilitation includes OT, PT, robotic arm training, acupuncture, and massage. Except for massage, all the training is boring, just repeating the same movements every day. (P06)  I have trained every weekday, only resting on weekends. My daughter had to push me in a wheelchair, and we are always rushing to get there on time; otherwise, I won’t be allowed to join the session. […] It’s exhausting. Sometimes my legs are swollen by the time I return to the ward. (P08) |
| Lack of continuity | At home, I can only go out for a walk and treat that as exercise. (P01)  After I went home, I didn’t train at all. I think many people are like this. How can you train at home? There’s no equipment, no one to guide you, and no conditions for proper training. (P03) |
| Perceived benefits | The therapists here are very experienced. The environment in the rehabilitation department is also heartwarming. Someone will hold my hand or greet me kindly with comforting words, this really touches me. (P02)  It’s been almost a month, and I’ll be discharged in a few days. My leg has improved a lot. (P03)  During the rehabilitation process, the doctors constantly encouraged me, saying things like ‘You’re making progress’ or ‘You’re much better than a few days ago.’ (P04) |
| **Unmet needs in the rehabilitation journey** | |
| Desire for better approaches | This is already the third hospital I have been to. I started at the best hospital in my province, then went to the best local rehabilitation center, and now I am here at what they say is the top rehabilitation center in the country. I don’t really know what treatment methods they use here, so I feel the need to try and find out. (P09) |
| Need for rehabilitation knowledge | We are not professionals, we don’t understand how this illness occurs or what the whole process involves. […] We truly lack knowledge. Now they are giving us these treatments, but we don’t know which ones are suitable for us. Even when doctors explain things, we still struggle to understand. (P10) |
| Demand for social respect | When I came to this hospital, I noticed that some of the staff members are also people with disabilities like us. It shows we can still work and support ourselves even if we are disabled. This encourages me not to give up on life or my future. This is unlike the negative comments made by some people in society. The doctors and nurses are all very kind; they never show any prejudice because of our condition. Their respectful communication really helps with psychological healing. (P19) |
| **Attitudes toward VR-based rehabilitation** | |
| Praised and positive perceptions | Once this device is fully developed and widely promoted, it could be placed at the nurses’ station for patients to use in rotation. Patients could even purchase one to use at home. (P03)  I think your VR device is very interesting—it’s completely virtual. My arm still feels a bit tired afterward, but the experience was enjoyable. […] Compared with the usual hospital treatments like cycling or traditional therapy, which are quite dull, this was much more engaging, though of course I can’t complain too much about hospital treatment either. (P20) |
| Skepticism and lack of interest | At my age, using computers is difficult. Sometimes I even feel dizzy. (P01)  It depends on how much your device costs. I just tried it and my first impression was good, but if the price is too high, not everyone will be able to afford it, right? (P10)  Honestly, I think what you are doing is great, but I just don’t think it’s necessary. No matter how good it is, I personally don’t need it. (P15)  I still prefer traditional rehabilitation. It feels more intuitive—you can see it and touch it. (P17) |
| **Recommendations for serious game design** | |
| Diversified game types | I think you should design more types of games. I prefer games set in natural scenery and am not interested in this sword-based game. (P16)  I think age differences matter. For people in their fifties or sixties, slicing fruit may not interest them. You could include chess or similar games that are more suitable for that age group. (P19)  There are gender differences. Women might prefer dancing games. (P20) |
| Customized training content | To me, muscle and strength training are most important because I want to walk independently. The game is great, and the virtual environment feels real, but I hope for more specialized training. For example, if I want to practice my hand, then exercises targeting finger movement or using chopsticks should be available. You could develop a game library with many options, and clinicians can select the ones that suit us. (P05) |
| Desired game functions | Is the vibration on the controller too weak? I didn’t feel any vibration. You could add prompts or voice cues like ‘please touch’ or use arrows such as ‘move the controller here.’ That would be very helpful. (P04)  I like a fully virtual environment because the scene feels realistic, and it gives you a sense of immersion. You’re not distracted by the outside environment, so you stay more focused during training. (P05)  If you add a scoring system—like getting 1000 points today and 1500 tomorrow—I would feel like I’m making progress. By the third day, I’d aim for an even higher score. That kind of thing motivates players. (P10)  Gradually increasing the difficulty, such as by speeding up or offering continuous stages, helps people adapt and stay interested in continuing. (P11) |
| **Suggested features of VR systems** | |
| Family involvement | This looks like a children's game, [...] can my grandson play it? (P01)  This is similar to the game consoles we have at home. Doesn’t Xbox have a game like this? […] I go home mainly to spend time with my daughter. She loves these kinds of games, so if we could play together, or if she could play with me in some way, that would be wonderful. (P21) |
| Access to personal rehabilitation data | Can you create a rehabilitation profile or progress tracking system? It looks like a game on the surface, but it’s actually rehabilitation training. You need to know what you’re training while playing and what you should do next. (P05) |
| Telerehabilitation support | If, during the rehabilitation process, doctors could see my progress and send me some rehabilitation-related suggestions, that would definitely be meaningful. (P02)  The equipment in hospitals is very expensive and too large to use at home. If VR can be applied to home-based rehabilitation, I think it’s an excellent method. It’s lightweight, you can use it at home, and it still provides effective rehabilitation. That’s very good. (P19) |
| Safety monitoring | You have to limit the game time. What if someone plays all night? That would be dangerous. (P12)  You could add some features focused on health monitoring. Since we mainly track blood pressure, you could include blood pressure measurements and provide specific values. That would make it medically useful and safer. Otherwise, someone might get too excited while playing and suddenly feel dizzy or faint. You could set an alert when blood pressure reaches a certain level, reminding the player to stop. (P19) |
| Technical support | If I buy it and take it home, I need help when the device has problems, something like a customer service number or a WeChat account. (P03)  If I am going to use it long-term, it needs to have warranty services. If it breaks, someone must be responsible for repairing it. (P12) |
